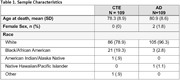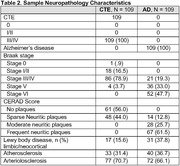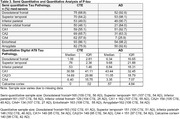# Cortical and subcortical p‐tau density in CTE: Comparison to Alzheimer’s disease

**DOI:** 10.1002/alz.092082

**Published:** 2025-01-09

**Authors:** Daniel Sconzo, Michael L. Alosco, Jenna R. Groh, Yorghos Tripodis, Daniel H. Daneshvar, Christopher Nowinski, Joseph N. Palmisano, Brett Martin, Jesse Mez, John F. Crary, Victor E. Alvarez, Bertrand Russell Huber, Ann C. McKee, Thor D. Stein, Jonathan D Cherry

**Affiliations:** ^1^ Boston University Chobanian & Avedisian School of Medicine, Boston, MA USA; ^2^ Boston University Alzheimer’s Disease Research and CTE Centers, Boston University Chobanian & Avedisian School of Medicine, Boston, MA USA; ^3^ Boston University Alzheimer’s Disease Research Center, Boston University Chobanian & Avedisian School of Medicine, Boston, MA USA; ^4^ Boston University Alzheimer’s Disease Research Center, Boston, MA USA; ^5^ Boston University School of Medicine, Boston, MA USA; ^6^ Framingham Heart Study, Boston, MA USA; ^7^ Boston University Chronic Traumatic Encephalopathy Center, Boston University Chobanian & Avedisian School of Medicine, Boston, MA USA; ^8^ Icahn School of Medicine at Mount Sinai, New York, NY USA; ^9^ Department of Pathology and Laboratory Medicine, Boston University Chobanian & Avedisian School of Medicine, Boston, MA USA; ^10^ Department of Pathology and Laboratory Medicine, Boston University School of Medicine, Boston, MA USA

## Abstract

**Background:**

The unique lesion of chronic traumatic encephalopathy (CTE) is the perivascular deposition of hyperphosphorylated tau at the depth of the cortical sulci. The distribution and molecular composition of p‐tau is distinct from Alzheimer’s disease (AD), but differential diagnostic challenges remain. Understanding disease differences in regional density of p‐tau will inform differential diagnosis and interpretation of in vivo biomarkers. Here, we compared autopsy‐confirmed CTE and AD across cortical and subcortical measurements of p‐tau density.

**Method:**

The sample included 109 brain donors with stage III/IV CTE, and 109 age (+/‐ 3 years) and sex similar brain donors with autopsy‐confirmed AD. While the original sample was similar, there is missing data across regions due to older cases not having AT8 stains. CTE was neuropathologically diagnosed using published criteria. NIA‐Reagan was used for AD. Neuropathologists used semi‐quantitative rating scales (0=none, 3=severe) to evaluate p‐tau severity in the dorsolateral frontal cortex (DLFC), inferior orbital frontal cortex (IFC), superior temporal cortex (STC), inferior parietal cortex (IPC), CA1, CA2, and CA4, entorhinal cortex (EC), and amygdala. Digital slide scanning of AT8 tissue was done for quantitative assessment of p‐tau density in the DLFC, STC, IPC, CA1, CA2/3, CA4, and calcarine cortex. Regression and analysis of variance models compared disease groups on each outcome, controlling for age.

**Result:**

On the semi‐quantitative rating scales, those with autopsy‐confirmed CTE had greater p‐tau severity in the CA4‐hippocampus compared with AD (OR=4.43, p=0.003); in contrast, AD had greater p‐tau severity in the CA1‐hippocampus (OR=0.07), EC (OR=0.10), and amygdala (OR=0.24) (ps<0.05). There were no differences for the CA2‐hippocampus. Regarding cortical regions, those with AD had greater p‐tau severity on the DLFC, IFC, STC, and IPC (ORs=0.03‐0.16, ps<0.05). A similar pattern was found for the quantitative p‐tau density data: CTE had greater p‐tau density in CA4‐ (marginal mean difference=7.45) and CA2/3 (marginal mean difference=9.86) (ps<0.01) and AD had higher density of p‐tau across the other regions (i.e., DLFC, IPC, STC, calcarine).

**Conclusion:**

Compared with AD, CA4‐hippocampus was most affected in CTE. AD had greater p‐tau density across cortical regions. Findings inform neuropathological differential diagnosis and interpretation of in vivo biomarkers in CTE and AD.